# Molecular characteristic of *Pasteurella multocida* isolates from Sumba Island at East Nusa Tenggara Province, Indonesia

**DOI:** 10.14202/vetworld.2020.104-109

**Published:** 2020-01-13

**Authors:** I. K. Narcana, I. W. Suardana, I. N. K. Besung

**Affiliations:** 1 Master Student of Veterinary Medicine, Faculty of Veterinary Medicine, Udayana University, Jl. PB. Sudirman Denpasar-Bali, 80232, Indonesia; 2 Department of Preventive Veterinary Medicine, Laboratory of Veterinary Public Health, Faculty of Veterinary Medicine, Udayana University, Jl. PB. Sudirman Denpasar-Bali, 80232, Indonesia; 3 Department of Pathobiology, Laboratory of Veterinary Microbiology, Faculty of Veterinary Medicine, Udayana University, Jl. PB. Sudirman Denpasar-Bali, 80232, Indonesia

**Keywords:** genetic relatedness, molecular genetic, *Pasteurella multocida*, Septicemia Epizootica, Sumba island

## Abstract

**Aim:**

This study aimed to determine the molecular characteristics of *Pasteurella multocida* isolates originated from Sumba Island, East Nusa Tenggara Province.

**Materials and Methods:**

The isolates of *P. multocida* stored in frozen storage were cultured in blood agar as a selective medium and identified conventionally. Molecular tests were initiated by DNA isolation and then followed by polymerase chain reaction tests with specific primers for the determination of *P. multocida* serotype A or B. Positive strain of serotype B was then confirmed molecularly using 16S rRNA gene primer and followed by the sequencing of nucleotides.

**Results:**

The study showed that both *P. multocida* isolates from Sumba island, i.e. PM1 is isolated from East Sumba district, while PM2 isolated from West Sumba district have 99.6% homology. Both isolates also known have 99% similarities with *P. multocida* originated from India, Britain, and Japan, respectively. The isolates share the same clade in the phylogenetic tree.

**Conclusion:**

The 16S rRNA sequencing revealed a high similarity of *P. multocida* serotype B:2 isolated from Sumba island with the Indian isolates although the sample size is very small. Therefore, further molecular studies like multilocus sequence typing, VNTR need to be performed using a larger number of samples to establish the genetic relatedness observed in this study.

## Introduction

*Pasteurella multocida* is a Gram-negative bacterium, which is the coccobacillus that normally lives on nasopharynx of animals [1,2]. It is also detectable in the gastrointestinal and urinary tracts [3]. The bacterium consists of several serotypes, and each serotype describes the nature of the disease. According to the Carter system, *P. multocida* is divided into five serotypes based on capsule antigen, namely, types A, B, D, E, and F. Furthermore, according to the Heddleston system with gel diffusion precipitin test, the bacteria are divided into 16 somatic antigen serotypes, namely, serotypes 1, 2, 3, 4, 5, 6, 7, 8, 9, 10, 11, 12, 13, 14, 15, and 16 [4,5].

As a normal flora in the upper respiratory tract, the agent can be pathogenic specifically if the body conditions of animals are decreasing. The germ of *P. multocida* will be pathogenic and causes several symptoms such as a decreasing of the appetite, weight loss, edema, and diarrhea and finally leads to death [6]. The bacteria are usually pathogenic in ruminants and poultries. Some diseases caused by *P. multocida* are fowl cholera in poultries; Septicemia Epizootica (SE)/Hemorrhagic Septicemia (HS) and Pasteurellosis Septicemia in cattle and buffaloes; pneumonia and Pasteurellosis Septicaemia in goats and sheep; and pneumonia, atrophic rhinitis, and septicemia in pigs [5].

The case of SE causes by *P. multocida* in Indonesia is one of the acute and fatal infectious diseases in ruminants, especially in buffaloes and cattle. The case is endemic and resulting in highly economical loss [7]. Moreover, Sumba island located in East of Nusa Tenggara Province is known as one of the areas, where the infection by this serotype is found every year. According to the surveillance conducted by the Animal Disease Investigation Center of Denpasar in 2014, there were identified 45 cases of SE in Timor Tengah Utara Regency, Province of East Nusa Tenggara, and the agent also has been identified recently. In Indonesia, the agent is usually confirmed conventionally by culturing the agent on selective medium and then classified based on morphology, carbohydrate fermentation, and serological tests [8]. Furthermore, genetic characterization as an accurate method to analyze the serotype of *P. multocida* [9] has not been performed yet.

This study was designed to confirm the conventional diagnosis and also to analysis of *P. multocida* from Sumba island molecularly. The study also pointed to find out the genetic relationship among *P. multocida* serotype B.

## Materials and Methods

### Ethical approval

The approval from the Institutional Animal Ethics Committee to carry out this study was not required due to no invasive technique was used.

### Bacterial isolates

Two isolates such as *P. multocida* as a result of 50 case samples tested from Sumba island, East Nusa Tenggara Province were used in this study. The isolates were preserved at Animal Disease Investigation Center in Denpasar with code PM B1 (isolate from East Sumba Regency) and PM B2 (isolate from West Sumba Regency), respectively. Both isolates were diluted with sterilized distilled water and then grown in blood agar media. The *P. multocid*a colonies, which were grayish-white color and 1.5 µm × 0.3 µm in diameter were stained with Gram’s staining and then observed microscopically. Identification was continuously confirmed by biochemical tests including catalase, mannitol, sucrose, H_2_S, and urease according to each of their standard procedures [10].

### DNA extraction

DNA from all isolates was extracted using QIAamp DNA Kits (cat. 51304) according to the manufacturer’s procedure with slight modification [11-13].

### Primers sets

Various sets of published primers (Table-1) were used for the molecular characterization of *P. multocida* in this study.

**Table-1:** The primers with their sequences used in the molecular characterization of *P. multocida* isolated from Sumba island.

Serogroups	Primer descriptions	Primer sequences	Annealing temperatures	Amplimer sizes (bp)
*P. multocida* serotype A	RGPM A5 RGPM A6	5’- AATGTTTGCGATAGTCCGTTAGA-3’ 5’- ATTTGGCGCCATATCGTC-3’	55°C	564
*P. multocida* serotype B	KTT 72 KTSP 61	5’- AGGCTCGTTTGGATTATGAAG-3’ 5’- ATCCGCTAACACACTCTC-3’	55°C	620
16S rRNA universal primer	B27 F U1492 R	5’- AGAGTTTGATCCTGGCTCAG-3’ 5’- AGAGTTTGATCCTGGCTCAG-3’	55°C	1502

*P. multocida*=*Pasteurella multocida*

### Polymerase chain reaction (PCR) amplification of *P. multocida* serotypes A and B

A 40 µl reaction mixture containing 2 μl DNA template (200 ng/μl), 34 μl PCR SuperMix 2×, and 2 μl (200 pmol/μl) of each forward and reverse primers for amplification of *P. multocida* serotypes A and B mentioned in Table-1 were prepared in this study. The amplification reaction was carried out with an initial denaturation at 94°C for 7 min, followed by 30 cycles at 94°C for 1 min, 55°C for 1 min, and 72°C for 2 min. The PCR reaction was ended with the final extension at 72°C for 5 min and then analyzed by electrophoresis in 1.5% agarose stained with ethidium bromide [14].

### PCR amplification of 16S rRNA gene of *Pasteurella multocida* spp.

The PCR program was carried out in 40 μl reaction volumes containing 2 μl DNA template (200 ng/μl), 34 μl PCR SuperMix 2×, and 2 μl (200 pmol/μl) of each primer 27F and U1492R (Table-1). The PCR amplification was performed according to the previous method [11,15] with initial DNA denaturation at 94°C for 5 min, followed by 35 cycles consisting of denaturation at 94°C for 1 min, annealing at 56°C for 1 min, and extension at 72°C for 1 min. Finally, the amplification was ended by a final extension at 72°C for 5 min. Furthermore, 5 μl of PCR product was analyzed by electrophoresis in 1% agarose [11,15].

### Sequencing and phylogenetic analysis

Sequencing of 16S rRNA gene of isolates was conducted using a genetic analyzer (ABI Prism 3130 and 3130xl Genetic Analyzer) at Eijkman Institute for Molecular Biology, Jakarta. The sequencing was used the similar primers with PCR reaction previously. The sequences were edited to exclude the PCR primer binding sites and they were corrected using MEGA 5.2 version software (https://www.megasoftware.net/). The full gene sequences were compared automatically using the BLAST program against the sequences of bacteria available in databanks (www.ncbi.nlm.nih.gov). The phylogenetic tree was constructed using the neighbor-joining algorithm method [16,17].

## Results

### Culturing of bacteria and biochemical test

**Figure-1:**
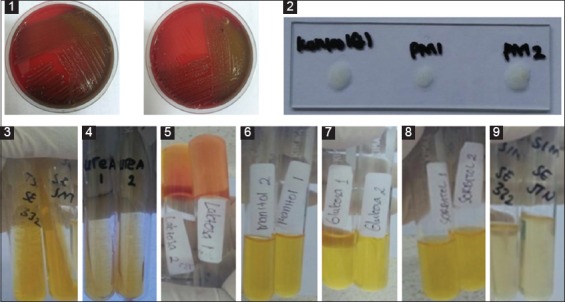
The growth results on blood agar media and biochemical tests of *Pasteurella multocida* isolates originated from Sumba island. 1: Blood agar; 2: Catalase test; 3:H_2_S test; 4: Urease test; 5: Lactose test; 6: Mannitol test; 7: Glucose test; 8: Sucrose test; 9: Indole rest.

The results of the study showed the growth of both isolates in blood agar media characterized by grayish-white colonies with diameter 1.5 µm × 0.3 µm size. The biochemical test showed that the isolates fermenting glucose, lactose, mannitol, sucrose, oxidase, and indole. The results of the test are shown in Figure-1.

**Figure-2:**
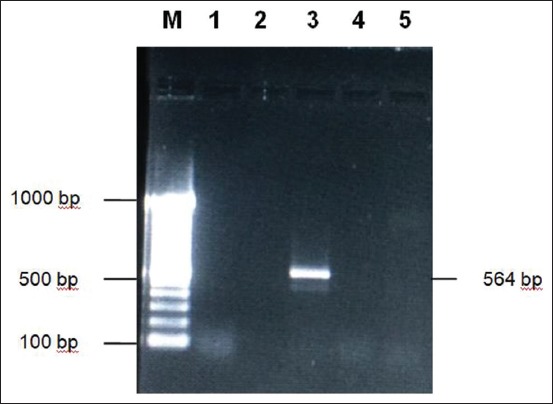
Amplification of *Pasteurella multocida* serotype A using RGPM A5 and RGPM A6 primers on 1% agarose. M: Marker 1 kb, 1: Isolate PM B1, 2: Isolate PM B2, 3: Positive control, 4: Negative control, 5: Negative control.

**Figure-3:**
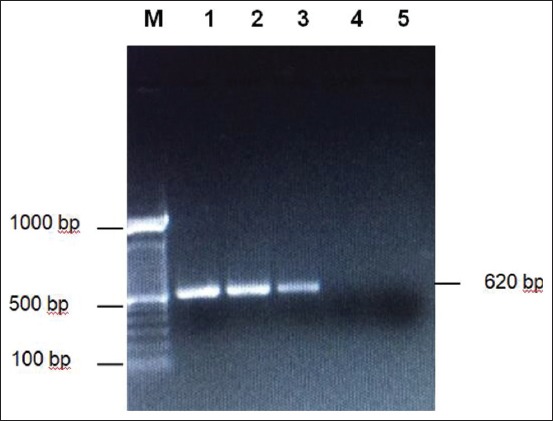
Amplification of *Pasteurella multocida* serotype B using KTT 72 and KTSP 61 primers on 1% agarose. M: Marker 1 kb, 1: Isolate PM B1, 2: Isolate PM B2, 3: Positive control, 4: Negative control, 5: Negative control.

The results in Figure-1 showed that *P. multocida* was negative hemolysis on blood agar medium, non-motile, fermented glucose, catalase, oxidase, and indole. Based on the biochemical tests above, both isolates PMB1 and PMB2 were positive *P. multocida* [8,18]. Furthermore, the molecular characterizations of both isolates are shown in Figures-2 and 3.

Figure-2 shows that the PM B1 and PM B2 isolates that were amplified using specific primers for *P. multocida* type A (RGPMA5 and RGPMA6) were negative. The PCR results did not show 564 bp fragments as shown in positive control. On the contrary, the isolates in Figure-3 which were amplified using specific primers *P. multocida* type B (KTT 72 and KTSP 61) show positive results characterized by PCR product 620 bp like a positive control. These results indicate that the *P. multocida* isolates from Sumba Island were *P. multocida* serotype B.

*P. multocida* serotype B is known as an acute and fatal agent, caused by SE on cattle and buffaloes [11]. The disease has been widely spread, especially in Southeast Asia and Africa. In general, there have been known two serotypes of *P. multocida*, namely, the B:2 as an Asian serotype and the E:2 as an African serotype [19].

**Figure-4:**
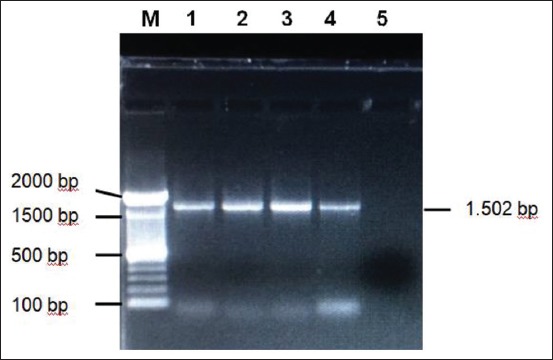
The results of amplification of the 16S rRNA gene of *P. multocida* isolates originated from Sumba Island on 1% agarose. M: Marker 1 kb, 1 and 2: Isolate PM B1, 3 and 4: PM B2, 5: Negative control.

In addition, the amplification of 16S rRNA gene as a universal method is mainly to find out the relationship both isolates to each other, which also shown positive results characterized by a single band in position 1502 bp (Figure-4).

**Figure-5:**
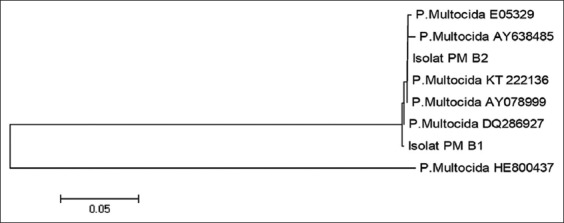
The phylogenetic tree of *Pasteurella multocida* Sumba isolates based on 16S rRNA gene sequences.

The PCR products of the 16S rRNA gene were sequenced and the nucleotide sequences were analyzed. The results of the alignment presented several variations. The nucleotides difference of *P. multocida* serotype B isolated from Sumba compare to others in the form of pairwise distances is shown in Table-2 while their phylogenetic tree is shown in Figure-5.

**Table-2:** The pairwise distance among the Pasteurella multocida isolated from Sumba Island compare to several nucleotide sequences accessed in GenBank.

	Isolate PM B1	Isolate PM B2	*P.multocide* KT 222136	*P.multocide* E05329	*P.multocide* HE800437	*P.multocide* AY078999	*P.multocide* DQ286927	*P.multocide* AY638485
Isolate PM B1								
Isolate PM B2	0.004							
*P.multocida* KT 222136	0.004	0.000						
*P.multocida* E05329	0.006	0.002	0.002					
*P.multocida* HE800437	0.512	0.512	0.512	0.517				
*P.multocida* AY078999	0.004	0.000	0.000	0.002	0.512			
*P.multocida* DQ286927	0.002	0.002	0.002	0.004	0.512	0.002		
*P.multocida* AY638485	0.009	0.004	0.004	0.006	0.526	0.004	0.006	

The results of the pairwise distances among *P. multocida* in Table-2 have shown that both *P. multocida* serotype B:2 from Sumba Island contained PM B1 originated from East Sumba Regency and the PM B2 originated from West Sumba Regency have high similarities to others. Furthermore, they have 99.6% similarities or only 4 of 1000 nucleotides are different from one to another. Further analysis comparing the nucleotide sequences in GenBank data, also shown a high similarity to others with the codes as follows: DQ286927 (Indian isolate), AY078999 (Britain isolate), KT222136 (Indian isolate), E05329 (Japan isolate), and AY638485 with percentages were 99.8, 99.6, 99.6, 99.4, and 99.1%, respectively. The results were contrary against the isolate with the code HE800437 (*P. multocida* isolates from Pakistan) with 48.8% similarities. The phylogenetic tree as a further analysis which was based on the data in Table-2 is grouping both local isolates to be one clade with DQ286927 (Indian isolate), AY078999 (Britain isolate), KT222136 (Indian isolate), E05329 (Japan isolate), and AY638485 isolate. The result of analysis also placed the isolate HE800437 from Pakistan in a different clade from the others (Figure-5).

## Discussion

Pasteurellosis has been recognized as a disease of major economic importance and confirmation of isolates which is difficult to solve, due to some multiple clinical symptoms and time-consuming laboratory procedures [20]. It has been proposed that the detection of *P. multocida* is greatly accelerated by the use of molecular technique. However, the advantages of molecular techniques if compared to the biochemical test including their high speed, sensitivity, specificity, and simplicity [14].

Based on the results of the study, the use of molecular techniques was very useful to classify *P. multocida* from Sumba island which has not been classified yet. Furthermore, using this molecular technique, serotyping of the *P. multocida* isolates from Sumba island will be quicker and more accurate. Hence, the results were in accordance with the previous study which showed that PCR technique was a rapid and reliable method to identify *P. multocida*. This method also provides a characterization in comparison with biochemical analysis and a conventional serotyping that may take up to 2 weeks [14]. Then, the use of specific primers KTT 72 and KTSP61 as to classify the serotype of *P. multocida* has been successfully also used by the researcher to identify the type B:2, B:5, and B:2,5 of *P. multocida* previously [21].

Another molecular technique such as the using of 16S rRNA gene as a target to classify and characterize the bacteria was also successful in this study. This method had been successfully used by the own researcher in analysis of *Escherichia coli* O157:H7 strains isolated from feces of human and Bali cattle [11,15]. In this matter, the use of 16S rRNA gene to analyze the genetic relatedness of *P. multocida* as an accurate and specifically technique also successfully used before by Dey *et al*. [22] In their study, they were analyzed 1468 bp fragments of 16S rRNA gene sequences which were compared against several isolates originated from cattle (PM75), pig (PM49), and sheep (PM82). In their research, they were found among isolates shared 99.9% homologies against a buffalo isolate (vaccine strain P52). Whereas, their similarities against the goat isolate (PM86) were found 99.8% homologies against the vaccine strain. The researcher was also found monophyletic against type B reference strain NCTC 10323 of *P. multocida* subsp. multocida. In their study, they were concluded that there are close relationships of HS causing *P. multocida* serotype B:2 isolates of buffalo and cattle with other uncommon hosts such as pig, sheep, and goat.

According to the theory, it is known that the use of 16S rRNA sequences is having several advantages such as numerous bacterial genera and species have been reclassified and renamed, the classification of uncultivable bacteria has been made possible, phylogenetic relationships have been determined, and the discovery and classification of novel bacterial species have been facilitated [23]. In addition, Patel [24] also reported, the use of 16S rRNA gene sequence to study the bacterial taxonomy has been used widely for some number reasons. These reasons include (i) its presence in almost all bacteria, often existing as a multigene family or operons; (ii) the function of the 16S rRNA gene overtime has not changed, suggesting that random sequence changes are a more accurate measure of time (evolution); and (iii) the 16S rRNA gene (1500 bp) is large enough for informatics purposes.

Based on the results of study, there were showed two isolates from Sumba island (PM B1 and PM B2 isolates) were confirmed having 99.8% similarities with *P. multocida* serotype B:2 from India. The high similarity of isolates is predicted in accordance with a history of the Ongole cattle which were farmed on Sumba island originated from India. Ongole cattle (*Bos indicus*) entering to Indonesia (Sumba Island) from the Madras region of Indian which was introduced by the Dutch East Indies Government in the early 20^th^ century or around 1906-1907. The Dutch Indies Government initiated the breeding of four types of cattle to Sumba island, namely, Bali cattle, Madura cattle, Javanese cows, and Ongole cattle. There are known only Ongole cows that can good adapt and develop rapidly among four types of cattle even though the Sumba island has a quite long dry season. Moreover, in 1914, the Dutch East Indies Government determined that Sumba island was the center for pure Ongole cattle breeding in Indonesia [25]. In this case, the researcher predicts, the entry of Ongole cows from India to Sumba island allows the agents of *P. multocida* which normally live in the upper respiratory tract of livestock to also be carried away.

Based on the limitation of sample size which was used in this study, the researcher suggests that the next study should be performed to clarify the genetic relatedness of *P. multocida* from Sumba isolates like the study has been conducted by Sarangi *et al*. In their study, they used multilocus sequence typing (MLST) technique as one of the best methods for long-term epidemiological study. Their results identified isolates from cattle circulating in India categorized as ST 122, ST 9, ST 229, ST 71, and ST 277 [26] so that the specification of *P. multocida* from Sumba Island can be clarified clearly.

The problem with the limitation of sample size in this study also be found as previously reported by other researchers in Indonesia. Pujiono *et al*. [27] just used three *P. multocida* isolates to identify and serogroup of *P. multocida* field isolates. In their study, they were combined the use of 16S rRNA test with other specific primers such as the primers to amplify the *kmt* gene and *bcbD* gene. By their combination, the three isolates were belonging to capsular serogroup B of *P. multocida*. Prihandini *et al*. [28] also used limitation isolates in their study. They were used five serotypes A, B, D, E, and F, with specific species primers (*kmt* gene) and specific primer for the amplification of capsular gene *hya*D-*hya*C and *bcb*D.

## Conclusion

The 16S rRNA sequencing revealed a high sixrity between *P. multocida* serotype B:2 isolated from Sumba island with the Indian isolates although the sample size is very small. Therefore, further molecular studies such as MLST and VNTR need to be performed using a larger number of samples to establish the genetic relatedness observed in this study.

## Authors’ Contributions

IKN, IWS, and INKB conceived and designed the experiments. IKN and IWS performed the experiments. All authors have read and approved the final manuscript.
